# Supplementary material for: Zinc–Cobalt Oxide Thin Films: High Curie Temperature Studied by Electron Magnetic Resonance
Source: Molecules. 2022 Dec 2;27(23):8500. doi: 10.3390/molecules27238500 (PMC9737795; doi:10.3390/molecules27238500)
Supplement: Supplementary file 1 [file molecules-27-08500-s001.zip › molecules-2028568-supplementary.pdf]

molecules

MDPI

Supplementary Materials

Zinc–Cobalt Oxide Thin Films: High Curie Temperature Studied by Electron Magnetic Resonance

Bogumił Cieniek [1,*], Ireneusz Stefaniuk [1], Ihor Virt [2,3], Roman V. Gamernyk [4] and Iwona Rogalska [1]

1 Institute of Materials Engineering, College of Natural Sciences, University of Rzeszow, Pigonia 1, 35-310 Rzeszow, Poland
2 Institute of Physics, College of Natural Sciences, University of Rzeszow, Pigonia 1, 35-310 Rzeszow, Poland
3 Institute of Physics, Mathematics, Economy and Innovation Technologies, Drohobych Ivan Franko State Pedagogical University, Stryiska 3, 82100 Drohobych, Ukraine
4 Department of Experimental Physics, Ivan Franko National University of Lviv, 79005 Lviv, Ukraine
* Correspondence: bcieniek@ur.edu.pl

List of Figures

Figure S1. SEM images of the $Zn_{0.8}Co_{0.2}O$/Ag film with clusters of Ag NPs.

Figure S2. XRD diffractogram of $Zn_{0.8}Co_{0.2}O$/Ag film on a silicon substrate.

Figure S3. EDS analysis of the $Zn_{0.8}Co_{0.2}O$/Ag composition.

List of Tables

Table S1. The average crystallite size of Ag NPs.

Table S2. EDS analysis of $Zn_{0.8}Co_{0.2}O$/Ag composites.

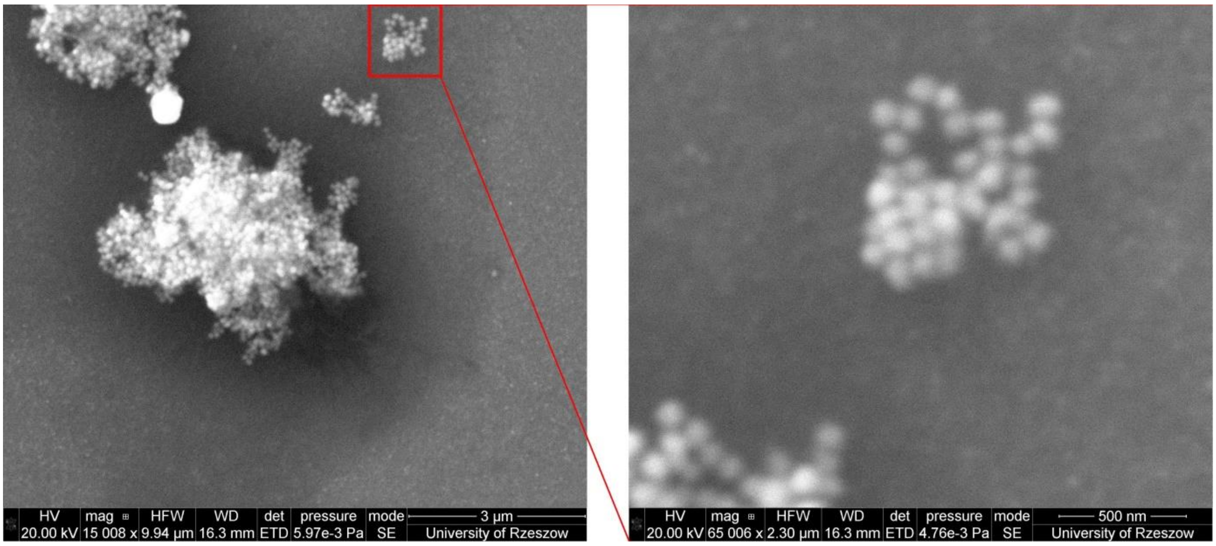

Figure S1. SEM images of the $Zn_{0.8}Co_{0.2}O$/Ag film with clusters of Ag NPs.

A SEM/FIB Quanta 3D 200i dual beam microscope was used to obtain the scanning electron microscope (SEM) images. SEM images of the $Zn_{0.8}Co_{0.2}O$ film embedded with Ag NPs are presented in Fig. S1. We observe clusters of silver nanoparticles, regularly

spread on the substrate. One of those clusters is shown on the left image, and the magnification of a single cluster is shown on the right image. The size of a single NP is about 80 nm.

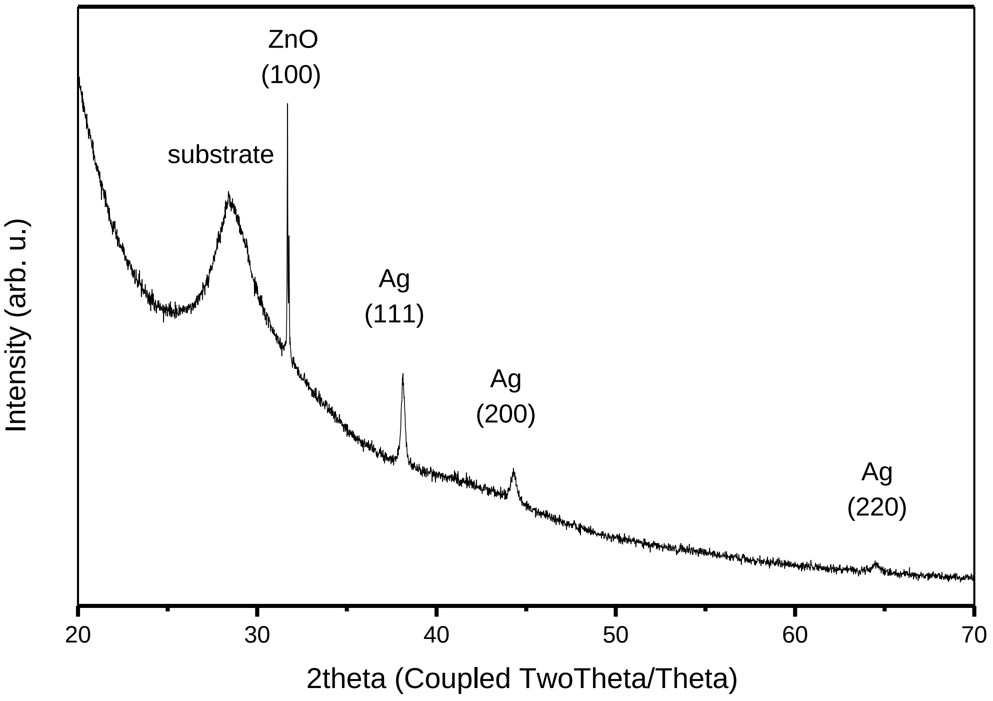

Figure S2. XRD diffractogram of $Zn_{0.8}Co_{0.2}O$/Ag film on a silicon substrate.

X-ray diffraction (XRD) measurements were performed on the XRD Bruker D8 Advance system with a Cu kα1 average 1.5418 Å, operated at 40 kV and 40 mA. XRD measurement was performed on the sample with the silicon substrate (Fig. S2). XRD results of metallic silver with dominant phase (111). The results also confirm that atoms of Ag are mainly form on the surface. The crystallite size was calculated from the XRD diffractogram using the well-known Debye-Scherrer formula:

$$d = \frac{K\lambda}{\beta\cos\theta}, \quad (1)$$

where K is Scherer constant (0.89), β is the full width at half maximum (FWHM), λ is the X-ray wavelength and θ is the Bragg diffraction angle. Using Equation (1) the average size of Ag NPs crystallites was determined to be 37.47 nm. Table 1 summarizes the results. Additionally, the crystallinity to amorphousness is 79.2% and 20.8%, respectively.

Table S1. The average crystallite size of Ag NPs.

No	Position 2θ (deg)	Plane name	Size (nm)
1	38.12	111	37.26
2	44.30	200	37.52
3	64.47	220	37.63

The difference between Ag crystallite measurements from the SEM image and the XRD diffractogram may be due to the fact that only one silver cluster is shown in the SEM image, and the XRD gives the result averaged over a larger area.

Figure S3 and Table S2 show the results of the EDS analysis. An Ag NPs cluster, shown in Figure S1, was selected for the location of the measurement.

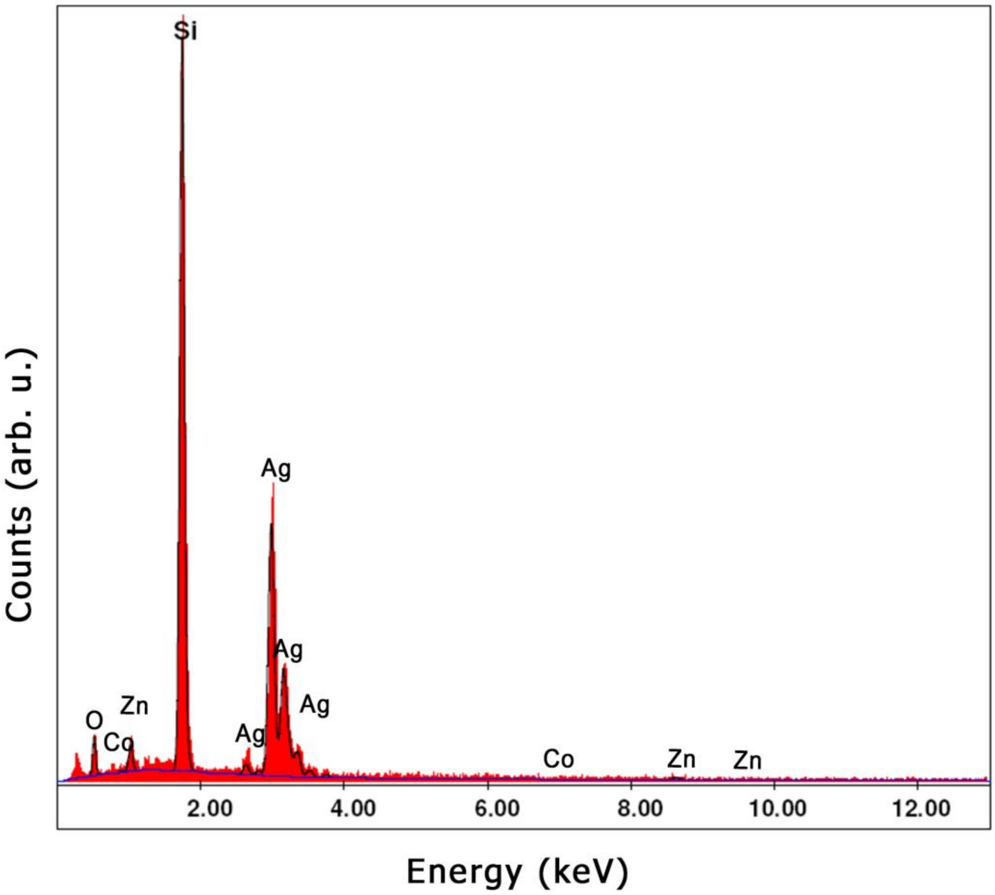

Figure S3. EDS analysis of the $Zn_{0.8}Co_{0.2}O$/Ag composition.

Table S2. EDS analysis of $Zn_{0.8}Co_{0.2}O$/Ag composites.

Elements (weight %)				
**OK**	**SiK**	**AgL**	**ZnK**	**CoK**
6.93	38.58	50.56	2.31	1.62
